# Fracture Mechanism and Toughness Optimization of Macroscopic Thick Graphene Oxide Film

**DOI:** 10.1038/srep13102

**Published:** 2015-08-27

**Authors:** Shibing Ye, Bin Chen, Jiachun Feng

**Affiliations:** 1 State Key Laboratory of Molecular Engineering of Polymers, Collaborative Innovation Center of Polymers and Polymer Composite Materials, Department of Macromolecular Science, Fudan University, Shanghai 200433, China

## Abstract

Combined high strength and toughness of film materials are rather important for their industrial applications. As a new class of films, graphene oxide films (GOFs) attract intense attention in many applications but are frequently divergent, inconsistent, and poorly reproducible in their mechanical properties. In this study, we first demonstrate that different chemical compositions and assembly structures probably are responsible for the difference in elongations between cast GOFs and filtration GOFs. Comprehensive analysis of the morphologies and mechanical properties indicates that the enhanced elongation of the thick cast GOFs is mainly attributed to the presence of a unique skin-wrinkles-skin structure, which more easily forms in cast GOFs than in filtration counterparts. On the basis of this finding, we attempt to optimize the strength-toughness performance of the cast GOFs by adjusting their structures. With an appropriate thickness of 12.5 μm, the GOFs can achieve an ultrahigh toughness up to 4.37 MJ m^−3^, which is even comparable to the polymer-toughening graphene/GO-based paper-like materials. Such an optimization of the mechanical properties from the perspective of skin-wrinkles-skin structure appears to be a universal approach that could be extended to a variety of other film materials.

The controlled assembly of nanoscale building blocks into macroscopic, ordered architectures is particularly valuable in nanoscience and nanotechnology. As a derivative of graphene, graphene oxide (GO) not only perfectly inherits the exceptional mechanical properties of graphene sheets, but also has high solubility and good processability^1^,^2^,^3^. Two-dimensional GO films (GOFs) provide a promising direction to translate the excellent properties of individual GO sheets into macroscopic materials at a manageable scale^4^,^5^,^6^,^7^. Since the pioneering work of Ruoff *et al.*^8^ on paper-like GOF, considerable efforts have been placed on the synthesis of GOFs^9^,^10^,^11^,^12^,^13^,^14^. These GOFs often show superior mechanical properties, such as high modulus (30–40 GPa) and tensile strength (60–150 MPa)^15^,^16^,^17^,^18^. Unfortunately, the toughness of pure GOFs is rather low (0.3–2.47 MJ m^−3^)^19^,^20^,^21^, which restricts their applications with adequate flexibility and stretch resistance, such as artificial muscle and filtration membranes. Although toughening GOFs with polymers could remarkably increase their ductility and toughness^22^,^23^,^24^, the introduction of polymers sometimes compromises the intrinsic biocompatibility and inevitably decreases the penetrability of pure GOFs^25^,^26^. For these reasons, achieving an extraordinary strength-toughness balance on pure GOFs is highly on demand. As shown in the summary statistics of the mechanical properties collected from the published papers (Fig. 1 and Table S1), it is worth noting that the GOFs prepared by cast drying (C-GOFs) generally possess higher elongation than those prepared by vacuum filtration (F-GOFs). Statistical result shows that most of C-GOFs exhibit high elongations over 1.5%^19^,^24^,^27^,^28^, while only one case among ten exceeds this value among the F-GOFs^12^,^16^. However, the origin responsible for this inexplicable difference between C-GOFs and F-GOFs is still not clear. It is therefore urgently needed to explore the underlying factors on the mechanical behaviors, the elongation in particular, of GOFs and build a relatively reliable framework for the correlation between structural features and mechanical properties of GOFs.

In this work, by comparatively investigating the structures and mechanical properties of F-GOFs and C-GOFs, we find that the difference in chemical composition and assembly structure between F-GOFs and C-GOFs might be responsible for the different elongations of the two types of GOFs. A unique skin-wrinkles-skin structure is more easily formed in the C-GOFs during cast process than in F-GOFs under vacuum pressure, which to a great extent improves the elongation at break of the C-GOFs. By adjusting the thickness, we obtained an optimized C-GOF with an extremely high elongation (9.8 ± 1.9%) as well as a high tensile strength (84.5 ± 9.8 MPa). The toughness reaches 4.37 ± 0.94 MJ m^−3^, which is among the best values for the reported graphene-based films. Our research suggests that the assembly structure is of great importance for the final mechanical properties of GOFs. In addition, the skin-wrinkles-skin model is expected to be a promising concept for the design of other high-performance paper-like materials.

## Results

### Comparison study of F-GOFs and C-GOFs

Note that several publications have already investigated the influencing factors and found that water content, GO size, and metal impurities greatly affect the final mechanical properties of GOFs^8^,^12^,^18^,^29^,^30^. From a different perspective, our study attempts to explore the potential impacts caused by different assembly techniques. The as-prepared GO (Figure S1) without further treatments was employed for both F-GOFs and C-GOFs, and the comparable levels of water content and interlayer spacings (Figure S2) for all the samples were controlled in order to avoid the inconsistency of data due to GO size and humidity. Briefly, C-GOFs were prepared by cast drying GO colloids (5 mg mL^−1^) on commercial PTFE plates, while the F-GOFs were prepared by vacuum filtration the same GO colloids (Figure S3 and S4). Subsequently, a long period of air drying was adopted until the moisture content approaches a comparable level. The thickness of GOFs could be well controlled from several microns to tens of microns by adjusting the volume of GO colloids.

Figure 2a shows the typical stress-strain curves of GOFs and Fig. 2b presents their average tensile strength and elongation. A thin F-GOF with a thickness of 4.9 μm has a tensile strength of 56.7 ± 2.3 MPa and an elongation of 2.5 ± 0.3%, which are similar to the results reported by Han *et al.*^12^. For the C-GOF with similar thickness, the strength changes to 59.6 ± 16 MPa with minor improvement, while the elongation increases to 3.2 ± 0.6% by ~31% compared with that of F-GOF. These values are well consistent with the results of the reported GOFs prepared by cast drying^19^,^28^. When the thickness of GOFs is increased to 9 ~ 10 μm, both the tensile strength and elongation of the thicker GOFs are slightly enhanced. The average tensile strength and elongation of the thicker F-GOF are increased to 60.9 ± 2.6 MPa and 3.9 ± 0.4%, respectively. With regard to the thicker C-GOF, these values raise to 62.6 ± 5.3 MPa and 4.2 ± 0.5%, respectively.

**Figure 1:**
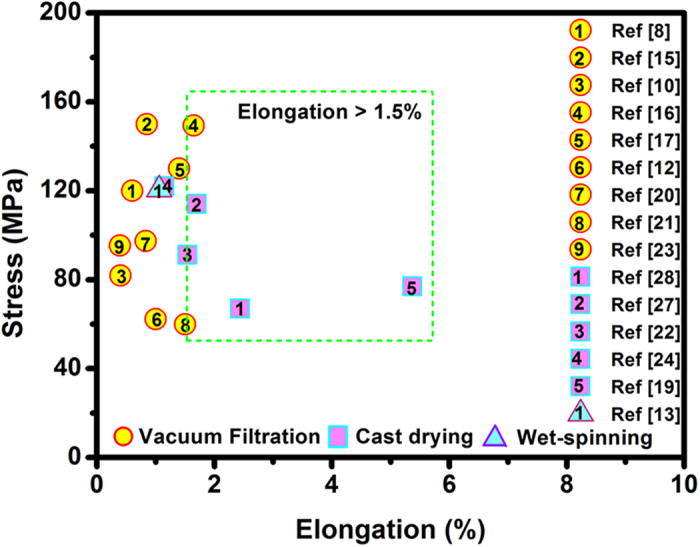
Statistical results of the mechanical properties of the reported pure GOFs.

**Figure 2:**
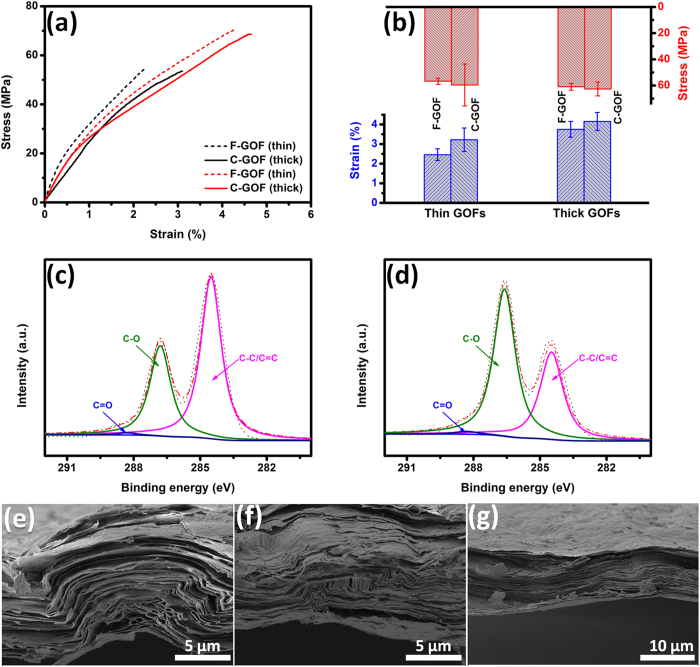
Typical stress-strain curves (**a**) and mechanical properties (**b**) of F-GOFs and C-GOFs in different thicknesses. XPS spectra of F-GOF (**c**) and C-GOF (**d**). SEM images cross-sections of the thick C-GOFs showing crumples (**e**), interlocks (**f**), and skins (**g**).

Comparison of the observed data of F-GOFs and C-GOFs indicates a good agreement with reported values (Fig. 1). Although the tensile strength is frequently fluctuating, the elongation of C-GOFs is consistently higher than that of F-GOFs. Detailed structural analysis was performed to understand the intrinsic factors affecting the elongation of GOFs. X-ray photoelectron spectroscopy (XPS) spectra were collected on the film surfaces unexposed to air. The C1s core level spectrum of the F-GOF can be fitted with three peaks at binding energies of 284.5 (63.5%), 286.8 (35.7%), and 288.3 eV (0.8%) which can be assigned to C=C sp^2^ bonds, C-O bonding, and C=O bonding, respectively (Fig. 2c). The C1s core level spectrum of the C-GOF was also fitted with three peaks at 284.5 (38.4%), 286.6 (60.4%), and 288.3 eV (1.2%) (Fig. 2d). It can be noted that the oxygen functionalities decrease from a total peak area of 61.6% for C-GOF to about 36.5% for F-GOF. Given the microstructure of the filter membrane (Figure S5), a large amount of nanoscale oxidation debris will undoubtedly be removed during vacuum filtration, which significantly decreases the quantity of oxygen moieties of GO^31^,^32^,^33^. According to the molecular dynamics simulations previously reported^7^,^29^, the GO with high O/C ratio has more hydrogen bonds than that with low O/C ratio at all hydration levels, due to the presence of more oxygen functional groups that can participate in the formation of hydrogen bonds. The morphologies of the fracture sections of GOFs were also examined by scanning electron microscope (SEM). F-GOF exhibits a compact, highly ordered, well-packed layered structure through the entire cross-section (Figure S6a), which looks like the microstructure of GOFs prepared using the same method reported by Ruoff *et al.* and Li *et al.*^8^,^15^. In comparison, C-GOF possesses a typical lamellar but relatively loose microstructure with apparent interlayer gaps (Figure S6b). We found that similar morphologies have also been observed in these C-GOFs reported by Cheng *et al.* and Liao *et al.*^22^,^28^. It appears that the evaporation-induced self-assembly of GO sheets during cast drying is relatively misaligned due to the lack of normal stress applied like in the vacuum filtration.

On the one hand, the removal of oxidation debris during filtration might lead to the reduction of hydrogen bonds in F-GOFs, which is probably one of the origins for the lower elongation of F-GOFs than C-GOFs. On the other hand, the interlayer gaps in C-GOFs could offer enough space for the realignments or slippages of GO sheets under stretching, which reasonably increases their elongations. Additionally, it is worth noting that the C-GOFs in our study easily copy the microscopic roughness of the PTFE surface (Figure S7a), producing a rough texture on the surface (Figure S7b). The resulting C-GOFs therefore show waved and corrugated sides (Figure S7c), in contrary to the straight and smooth sides of the F-GOFs (Figure S7d). Interestingly, these ripples can offer additional ductility^19^. On the basis of these results, it is assumed that the enhanced elongation of C-GOFs than F-GOFs should be attributed to the synergetic effect of the different chemical compositions and assembly structures. In this experiment, when other factors kept constant, the cast drying demonstrates more potentials than vacuum filtration as an assembly technique in preparing GOFs with large elongations. This phenomenon seems consistent with the published results. To further understand the nature of the improved elongation, we focus on the C-GOFs in terms of their formation and fracture process.

### Formation and fracture mechanism of C-GOFs

**Figure 3:**
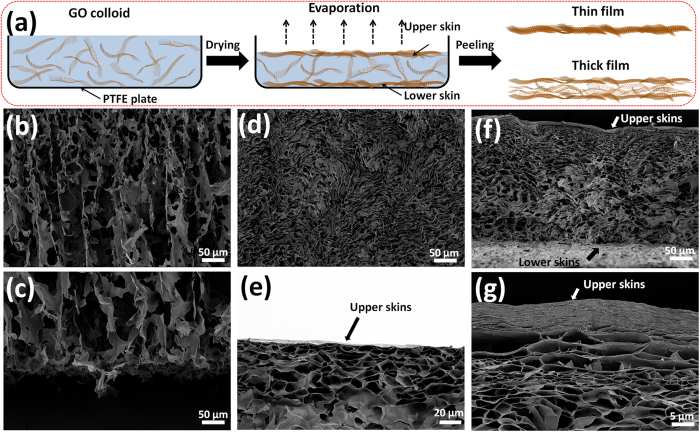
(**a**) Schematic illustration for the formation process of C-GOFs. SEM images of the cross-sections of the freeze-dried GO gels at different stages: (**b,c**) initial 5 mg mL^−1^ GO colloids, (**d,e**) gels after drying for 1 day, and (**f,g**) gels after drying for 3 days.

Interestingly, a comparison of the mechanical properties of C-GOFs with different thicknesses indicates that the thicker film exhibits a relatively larger elongation than the thinner one. Complementarily, the cross-sections of the 10.5-μm-thick C-GOFs were carefully observed by SEM. In addition to the waves on the surface, more interlayer gaps, quite a few GO crumples (Fig. 2e) and interlocked structures (Fig. 2f) could be found. More interestingly, these wrinkled regions are covered by two thin but compact GO skins (Fig. 2g), which might be initially formed through evaporation and sedimentation^19^. A possible formation process is proposed as schematically illustrated in Fig. 3a, while SEM images of GOFs at different drying stages were provided in Fig. 3b–g. GO colloid initially exhibits a loose, homogeneous GO network (Fig. 3b), without any observable skin-like structures (Fig. 3c). The intrinsic amphiphilicity of GO sheets endows them with a strong tendency for surface enrichment at the liquid/air interface^2^,^34^. With the water spilling out from the colloid during air drying, there are increased possibilities for GO sheets to collide and interact with each other and to move up to the liquid/air interface^35^. This gradually produces a dense GO network (Fig. 3d) and a compact upper skin (Fig. 3e). Meanwhile, the sedimentation of GO sheets at the solid/liquid interface also produces a lower skin, which is a little different from the upper one (Figure S8). The limited mobility of the GO sheets within the two skins hinders their parallel alignment^24^, which leads to relatively misaligned and wrinkled constructions. As the evaporation further continues, the GO networks in the concentrated gels collapse and aggregate, generating a hierarchical, wrinkled structure with many crumples and interlocks (Fig. 3f,g). As a consequence, a very apparent skin-wrinkles-skin structure could be always observed in the thick GOFs (Figure S9). Obviously, the proportion of wrinkled part in the C-GOFs will increase with film thickness. It should also be noted that, with increasing the volume of GO colloids, the filtration process is easily hindered due to the initial deposition of GO sheets onto membrane, which makes the assembly of the remaining GO colloids in a way more like evaporation. Specially, the concentration of GO colloids (5 mg mL^−1^) in this study is much higher than that used in other works^8^,^10^,^15^, which further slowdown the filtration process. The skin-wrinkles-skin structure could be observed occasionally in the thick F-GOFs.

**Figure 4:**
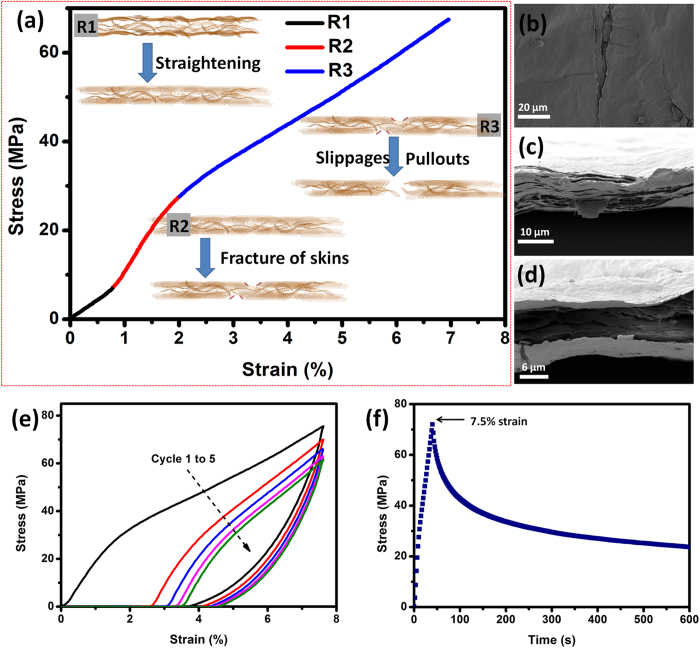
(**a**) A selected stress-strain curve of the 13.8-μm-thick C-GOF, demonstrating the fracture mechanism of the thick C-GOFs. SEM images of (**b**) the ruptured skins, and (**c,d**) irregular fracture sections of the stretched GOFs. (**e**) Loading-unloading cycling and (**f**) relaxation test of the 13.8-μm-thick C-GOF within 7.5% strain.

As shown in a selected stress-strain curve of a specific thick C-GOF (Fig. 4a), nearly three regimes of deformation, R1, R2, and R3, could be observed. According to the skin-wrinkles-skin model of C-GOFs, these three failure modes can be explained as follows. R1, 0 ~ 0.8%, possibly corresponds to the initial straightening of the waves and ripples on the GOF surface upon the tensile loading, which is quite small and sometimes indiscernible under accidental high prestress. R2, 0.8 ~ 2%, possibly corresponds to the fracture of the two compact GO skins (Fig. 4b and Figure S10), exhibiting high Young modulus and a similar behavior to the highly ordered F-GOFs. R3, 2 ~ 8%, possibly corresponds to the slippages and pullouts of the GO sheets, producing irregular fracture sections (Fig. 4c,d and Figure S11). Notably, the R3 regime, that is strongly associated with the wrinkled part of C-GOF, makes a great contribution to its total elongation. Based on this proposed fracture mechanism, first, the interlocked structures provide more interfacial area available for stress transfer between GO layers, increasing the ultimate tensile strength to a certain degree. Second, the abundant crumples and gaps allow the realignments and facilitate the slippages of GO sheets under external loading.

In order to further confirm the skin-wrinkles-skin model and fracture mechanism, tests of loading-unloading cycling and stress relaxation were conducted on the above thick C-GOF. As shown in Fig. 4e, the first loading-unloading curve within a high strain (~7.5%) reveals an irreversible deformation of ~4%. The other four cyclic loading-unloading curves have better repeatability, producing smaller and smaller permanent deformations. These results indicate that the crumpled structures could be stretched and the interlocked structures could be broken upon repeated loading^24^, but these structures could not be completely destroyed without further loading. The stress relaxation test in Fig. 4f reveals that the stress of C-GOF under 7.5% strain gradually decreases with the time, implying the realignments or slippages of the neighboring GO sheets. Results of both tests prove the presence of R3 regime during the tensile process of thick C-GOFs.

### Optimization of the toughness of C-GOFs

**Figure 5:**
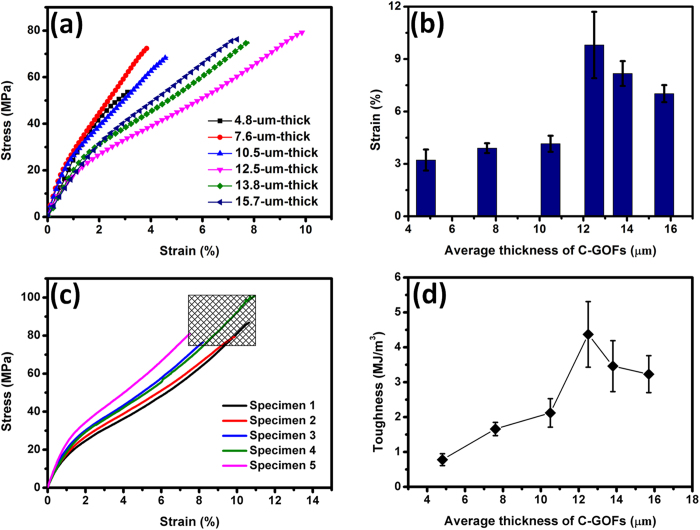
(**a**) Typical stress-strain curves and (**b**) average elongations of the C-GOFs in different thicknesses. (**c**) Five stress-strain curves of the 12.5-μm-thick C-GOF obtained from different specimens. (**d**) Thickness dependence of the toughness calculated from the stress-strain curves of C-GOFs.

The skin-wrinkles-skin model reasonably explains the improved elongation of our prepared C-GOFs. In this premise, a C-GOF with optimal strength-toughness balance can be conceived. Figure 5a shows the typical stress-strain curves of a series of C-GOFs in various thicknesses (Figure S12) and the detailed mechanical properties are listed in Table S2. With increasing the thickness of C-GOFs from 4.8 to 15.7 μm, the tensile strength does not change much with a value around 70 MPa. In contrast, the elongations belonging to R3 regime in the stress-strain curves vary with the thickness of C-GOFs, while the R1 and R2 regimes nearly have identical elongation, which are consistent with the skin-wrinkles-skin model of C-GOFs and their fracture mechanism. The elongations at break are tested to be 3.2 ± 0.6, 3.9 ± 0.3, 4.2 ± 0.5, 9.8 ± 1.9, 8.2 ± 0.7, and 7.0 ± 0.5% for the 4.8-, 7.6-, 10.5-, 12.5-, 13.8-, and 15.7-μm-thick C-GOFs, respectively (Fig. 5b). It is obvious that the elongation gradually increases as C-GOFs’ thickness increases from 4.8 to 12.5 μm. However, no more increase in the elongations was observed if further increasing the thickness to 13.8 or 15.7 μm. Based on our proposed fracture mechanism, it is speculated that the ordered skin structures take up a dominant part in the thin C-GOF with thicknesses lower than 12 μm, which leads to a skin-fracture-dominated way. As a result, these C-GOFs exhibit a mechanical behavior similar to the highly ordered F-GOFs, which generally possess relatively low elongations. For the thicker C-GOFs with thicknesses more than 12 μm, they tend to follow a wrinkles-fracture-dominated way upon loading, thus producing larger elongations. However, more aggregates of GO sheets, heterogeneities, and defects will inevitably occur in the film with further increasing the thickness. This might be why the elongation does not rise proportionately with the thickness above 12 μm.

Among all the investigated C-GOFs, the 12.5-μm-thick C-GOF exhibits a best mechanical performance with a high stress (84.5 ± 9.8 MPa) and a large strain (9.8 ± 1.9%) (Fig. 5c). Although the tensile strength is lower than the highest value reported by Wallace and Li *et al.*^15^, the elongation at break far exceeds those of reported F-GOF (2.3%)^12^, reduced GO (rGO) paper^36^, and rGO/MoS_2_ paper^37^, and the record-high value of the C-GOF (5.4%) previously reported (Figure S13)^19^. This ultrahigh elongation should be attributed to the synergetic effect of the ideal skin-wrinkles-skin structure and an appropriate amount of interlamellar water, which serves as efficient lubricant for the slippages of GO sheets. The additional experiments confirm that the annealing of C-GOFs at higher temperatures slightly decreases their elongation and enhances the tensile strength (Figure S14).

**Figure 6:**
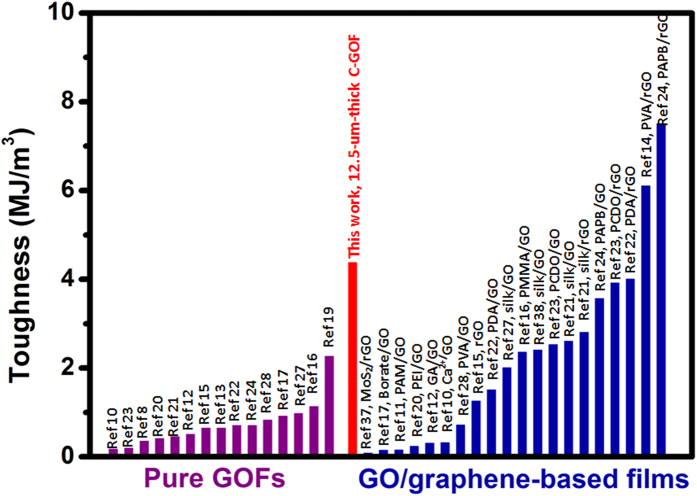
Comparison of toughness of our optimized C-GOF with the pure GOFs and modified GO/rGO-based paper-like materials published in the literatures.

As shown in Fig. 5d, the volumetric toughness is calculated to be 0.78 ± 0.17, 1.66 ± 0.19, 2.12 ± 0.41, 4.37 ± 0.94, 3.46 ± 0.73, and 3.23 ± 0.53 MJ m^−3^ for the 4.8-, 7.6-, 10.5-, 12.5-, 13.8-, and 15.7-μm-thick C-GOFs, respectively. The obtained highest toughness (4.37 ± 0.94 MJ m^−3^) of the 12.5-μm-thick C-GOF is higher than those found in the reported filtration GOF (1.24 MJ m^−3^)^16^ and cast GOF (2.26 MJ m^−3^)^19^, and is the highest value among the pure GOFs, which is even comparable to those of the modified graphene-based films to the best of our knowledge (Fig. 6 and Table S3)^22^,^23^,^24^,^38^. It is worth noting that this value is still much lower than the extremely high toughness (17 MJ m^−3^) of the fibers prepared by scrolling C-GOFs^19^.

## Discussion

Generally, the desirable mechanical properties of GOFs are obtained with the mediation of a small number of intercalated water molecules (~5–15 wt%), which produce numerous hydrogen bonds between adjacent layers in the paper structure^7^,^29^. Therefore, the proper water content is essential when optimizing the mechanical properties of GOFs by adjusting their structure. Another point should not be ignored is that the initial GO colloid in this study never underwent extensive sonication that always occurs in other reports. It has been proven that sonication of 0.5 h could significantly break the original large GO sheets into rather small pieces, which undermines the final mechanical properties of the resulting nanocomposites^26^. So the unique skin-wrinkles-skin structure together with a proper amount of water and high GO quality leads to a high toughness for the C-GOFs with slightly compromising the tensile strength. Actually, it is difficult to achieve both record-high strength and toughness for a multicomponent material due to the conflicting reinforcing mechanisms.

GOF is known to be a rather complicated system due to the numerous underlying factors that possibly affect their microstructures and mechanical properties^12^,^29^,^30^,^39^. That is why various fracture mechanisms of GOFs were proposed for their rationality and applicability^8^,^10^,^23^,^24^. Strictly speaking, the skin-wrinkles-skin structure is a specific fracture mechanism for these C-GOFs with a certain thickness, but not applicable everywhere. Despite the thickness dependence of the mechanical properties of the GOFs, we note that the thickness increments studied in this work was probably too coarse to catch the optimal thickness. Further work is needed to achieve more quantifiable design and prediction of the mechanical properties of GOFs. Our results suggest that for the optimization of the GOFs, assembly structure, water content, and GO quality must be taken into account.

## Methods

### Synthesis of GO

GO was prepared by the method reported by Marcano *et al.*^40^ with a slight modification. Typically, graphite (5 g, Shengtai Graphite Company, P. R. China) was dispersed with magnetic stirring in a mixture of H_2_SO_4_ (200 mL) and H_3_PO_4_ (40 mL) in an ice bath. Then KMnO_4_ (25 g) was slowly added and dissolved within 30 min. After that, the ice bath was removed, and the oxidation of graphite proceeded in a 40 ^o^C-bath for 4 h. Next, the cooling black slurry was poured slowly into cold water (600 mL) containing 40 mL of 35% H_2_O_2_. Finally, the GO bright yellow dispersion was left overnight to allow for complete neutralization of KMnO_4_. To purify the GO, the supernatant of the yellow GO dispersion was decanted, and the GO precipitate was dispersed in 5 wt % H_2_SO_4_ (600 mL) followed by centrifugation at 8000 rpm for 10 min (3×). Similar redispersion of GO precipitate in deionized water was also conducted followed by centrifugation at 10000 rpm for 20 min (3×). The washed GO slurry was then dialyzed in ample deionized water for 7 days until the pH reaches 6 ~ 7. Large unexfoliated graphite particles that might be present were removed by centrifugation at 4000 rpm for 5 min. In order to preserve the large sheet size, sonication of GO colloids during purification is avoided. The resulting GO was stored in the form of never-dried colloids with a high concentration of ~12.5 mg mL^−1^.

### Fabrication of GOFs

F-GOFs were prepared by filtration of GO colloids (5 mg mL^−1^) through a mixed cellulose esters filter membrane (47 mm in diameter, 0.22 μm in pore size) under vacuum pressure. C-GOFs were prepared by pouring GO colloids (5 mg mL^−1^) into the PTFE plates (Shanghai Daitao Rubber&Plastic Products Co., Ltd.), followed by air drying at room temperature for 7 days. The condition experiments confirmed that 7-day air drying could ensure the mechanical properties of GOFs reached a steady state (Figure S15). The thickness of GOFs varies between 4 and 16 μm by controlling the volume of GO colloids.

### Characterization and tests

Cross-sections of GOFs were obtained by fracturing samples in liquid nitrogen. The fractured surfaces and interfaces of GOFs were collected from the samples subjected to tensile experiments. The transition structures of GOFs at different stages during the formation process were obtained by freeze-drying the GO colloids and gels at −50 ^o^C and less than 20 Pa for 3 days. The morphologies of all above samples were characterized using a field-emission SEM (Zeiss Ultra 55, German) at an acceleration voltage of 3 kV. XPS spectra of the GOFs’ surfaces were recorded on a Thermo ESCALAB 250XI electron spectrometer with monochromatic 150 W Al Kα radiation. Mechanical properties were measured using a universal testing machine (SANS CMT-6503, Shenzhen, China), fitted with a 50 N load cell, at a loading rate of 1 mm min^−1^. All samples were cut into strips with a length of 20 mm and a width of 4 mm using razor blades. GOFs strips were covered by sandpapers to enhance the friction between samples and grips before testing. (Figure S16). At least five tests were performed for each sample, from which the mean values and standard deviations were derived.

## Additional Information

**How to cite this article**: Ye, S. *et al.* Fracture Mechanism and Toughness Optimization of Macroscopic Thick Graphene Oxide Film. *Sci. Rep.* **5**, 13102; doi: 10.1038/srep13102 (2015).

## Supplementary Material

Supplementary Information
