# Supplementary material for: Fracture Mechanism and Toughness Optimization of Macroscopic Thick Graphene Oxide Film
Source: Sci Rep. 2015 Aug 27;5:13102. doi: 10.1038/srep13102 (PMC4550846; doi:10.1038/srep13102)
Supplement: Supplementary Information [file srep13102-s1.pdf]

Supplementary Information for

Fracture Mechanism and Toughness Optimization of Macroscopic Thick Graphene Oxide Film

Shibing Ye[#], Bin Chen[#], and Jiachun Feng*

State Key Laboratory of Molecular Engineering of Polymers, Collaborative Innovation Center of Polymers and Polymer Composite Materials, Department of Macromolecular Science, Fudan University, Shanghai 200433, China.

#These authors contributed equally.

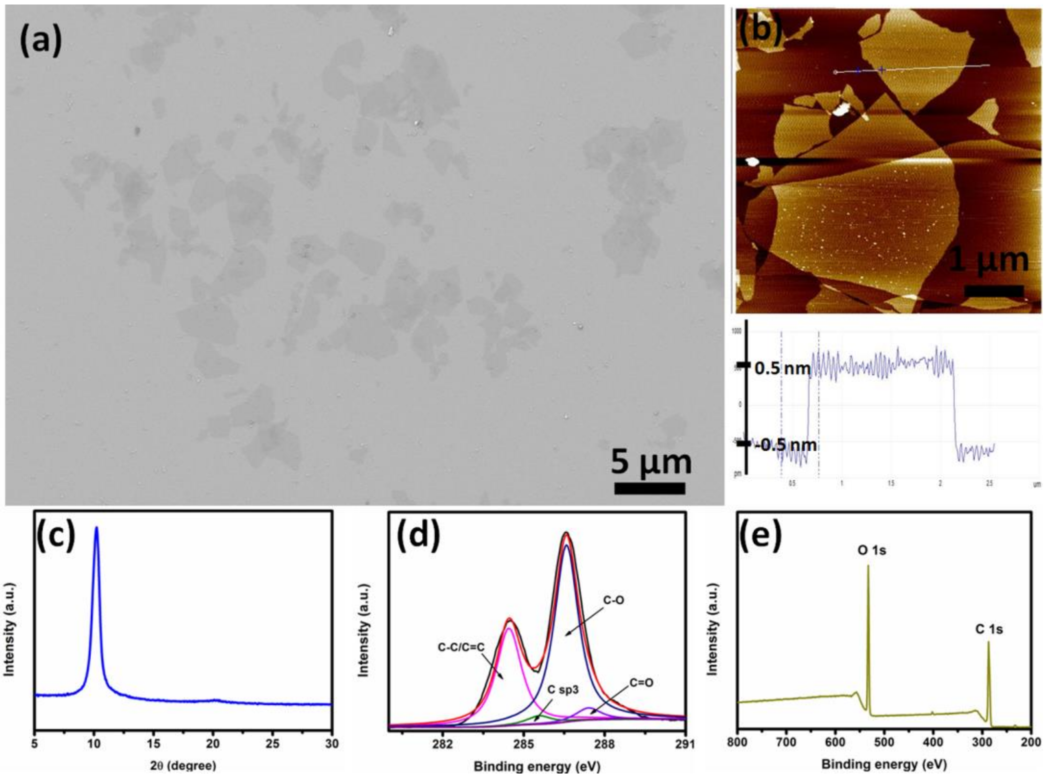

Figure S1 Structural characterization of GO. (a) SEM image, (b) AFM image, (c) XRD pattern, and (d-e) XPS spectra of GO used in this work. The morphology and structural analysis indicates GO sheets are completely exfoliated with a thickness ~1 nm and a C/O ratio of ~1.8.

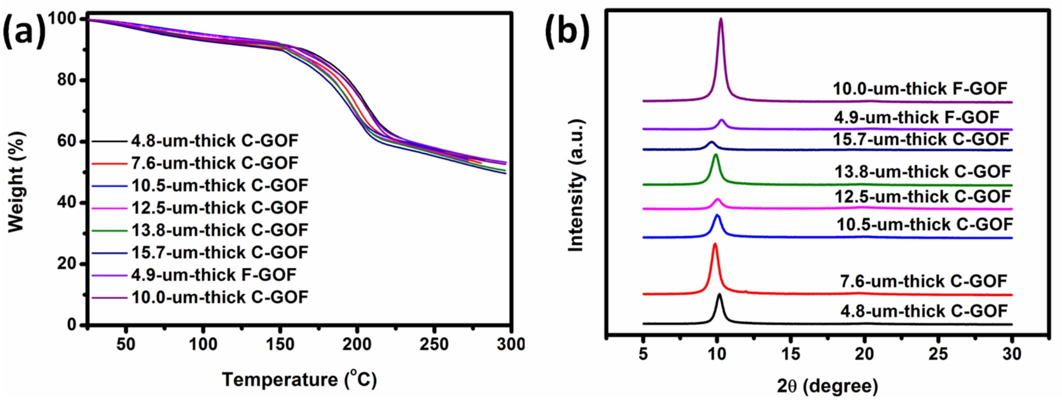

Figure S2 TGA curves (a) and XRD patterns (b) of our prepared F-GOFs and C-GOFs. TGA curves that obtained by heating GOFs from room temperature to 300 °C with a rate of 10 °C/min under a nitrogen atmosphere. It is obvious to find that these TGA curves almost retrace each other, especially before 150 °C, which indicates similar weight loss behaviors. Here, we used the weight loss before 150 °C to quantificationally determine the water content in GOFs. Although these values are different, they are controlled at comparable levels around 8.6±0.4%. There is not apparent difference in the lamellar ordering for all the C-GOFs in different thicknesses. This indicates similar interlayer spacing of C-GOFs, which is consistent with the similar water content. Note that the interlayer spacing of F-GOFs are found to be smaller than those of C-GOFs, which might be attributed to that the removal of the oxidation debris during the filtration.

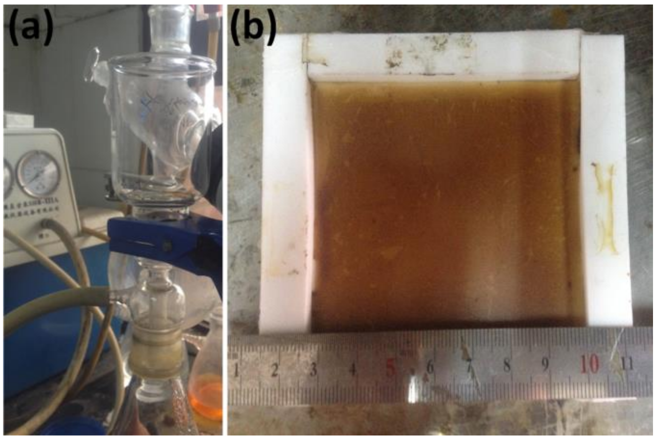

Figure S3 Digital pictures of filter (a) and PTFE plate (b).

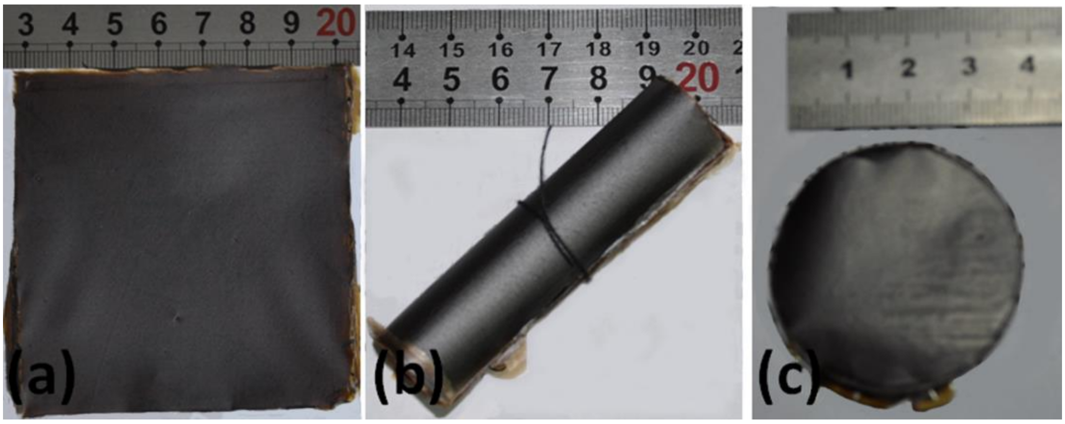

Figure S4 Digital pictures of (a-b) C-GOF, and (c) F-GOF.

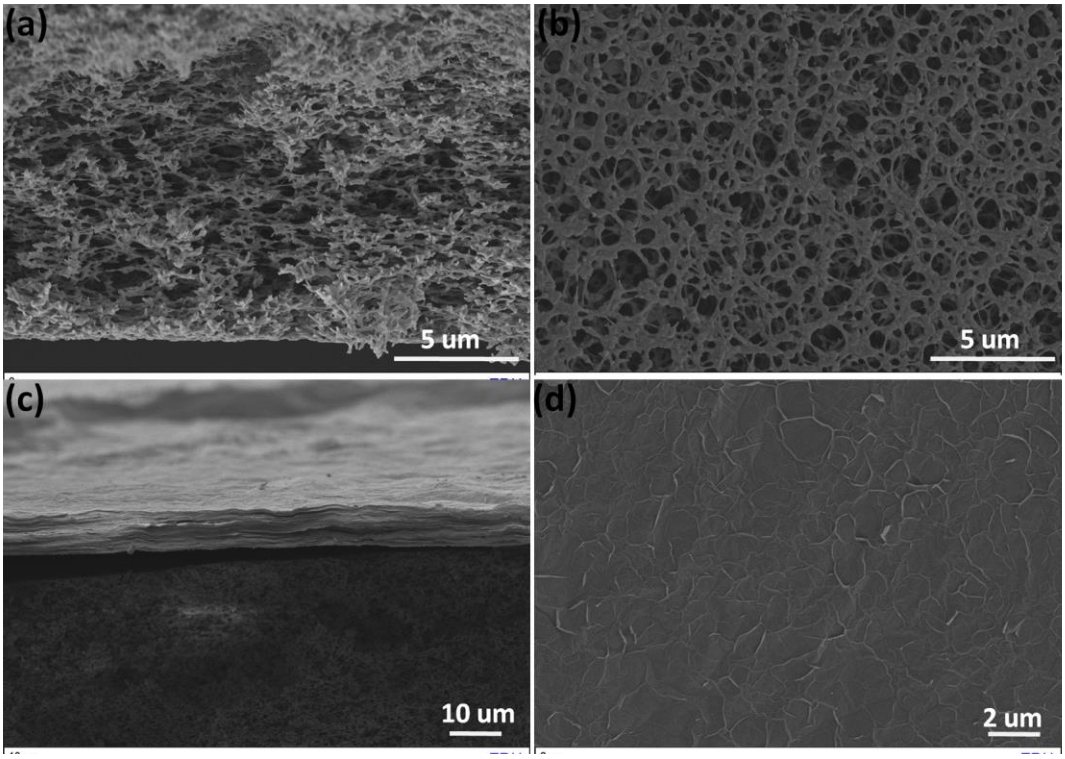

Figure S5 SEM images of the cross-section (a) and surface (b) of the filter membrane, cross-sections of F-GOF on the filter membrane (c) and the surface of F-GOF contacted with filter membrane (d).

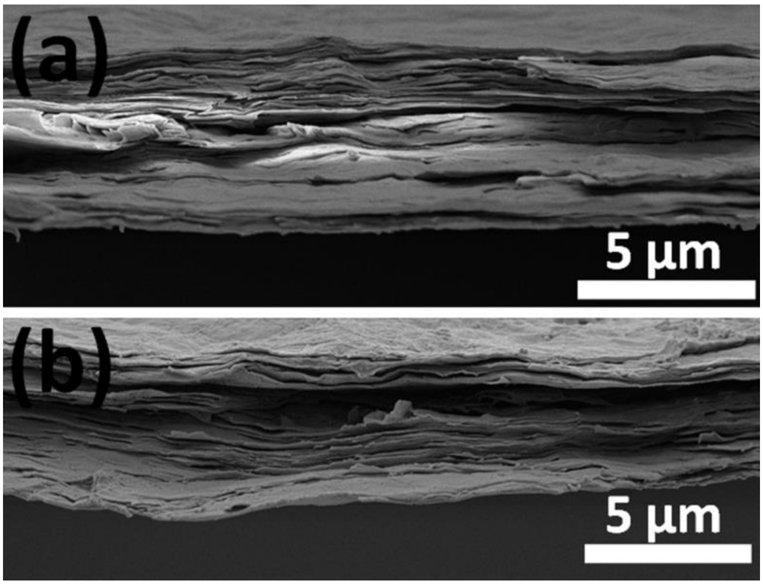

Figure S6 SEM images of the cross-sections of 4.9-µm-thick F-GOF (a) and 4.8-µm-thick C-GOF (b).

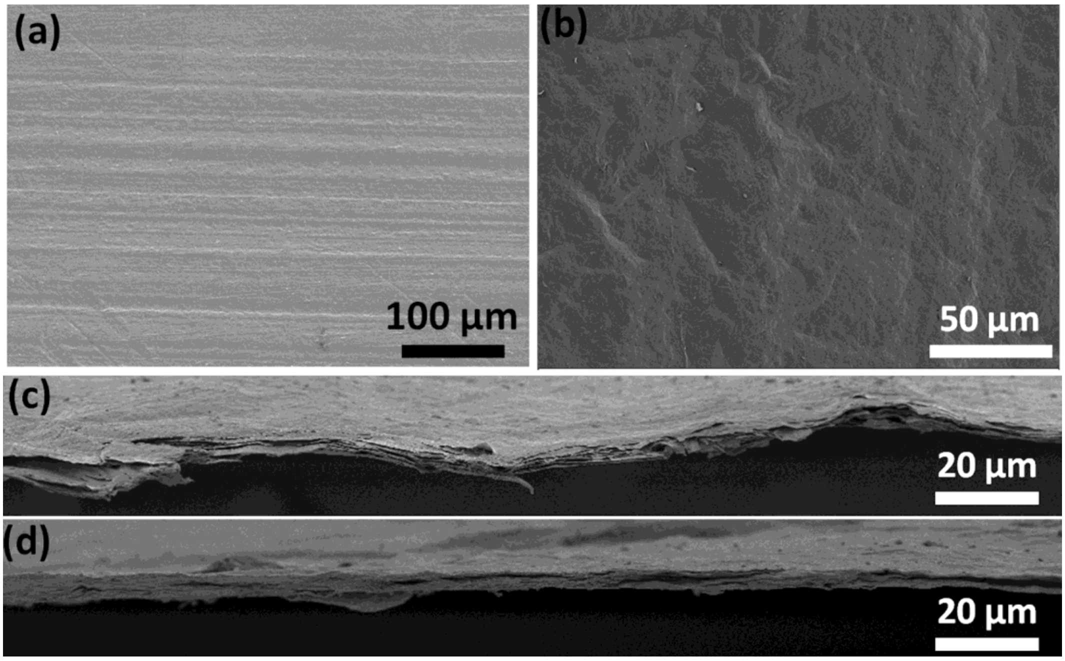

Figure S7 SEM images of the roughness of the PTFE surface (a), rough surface of the C-GOF (b), waved sides of C-GOF (c) and F-GOF (d).

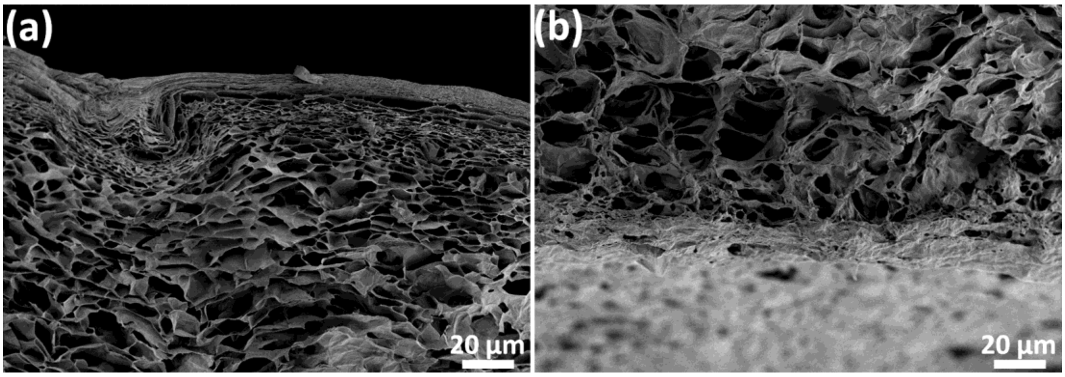

Figure S8 SEM images of the formed upper skin (a) and lower skin (b) of the GOFs during the air drying.

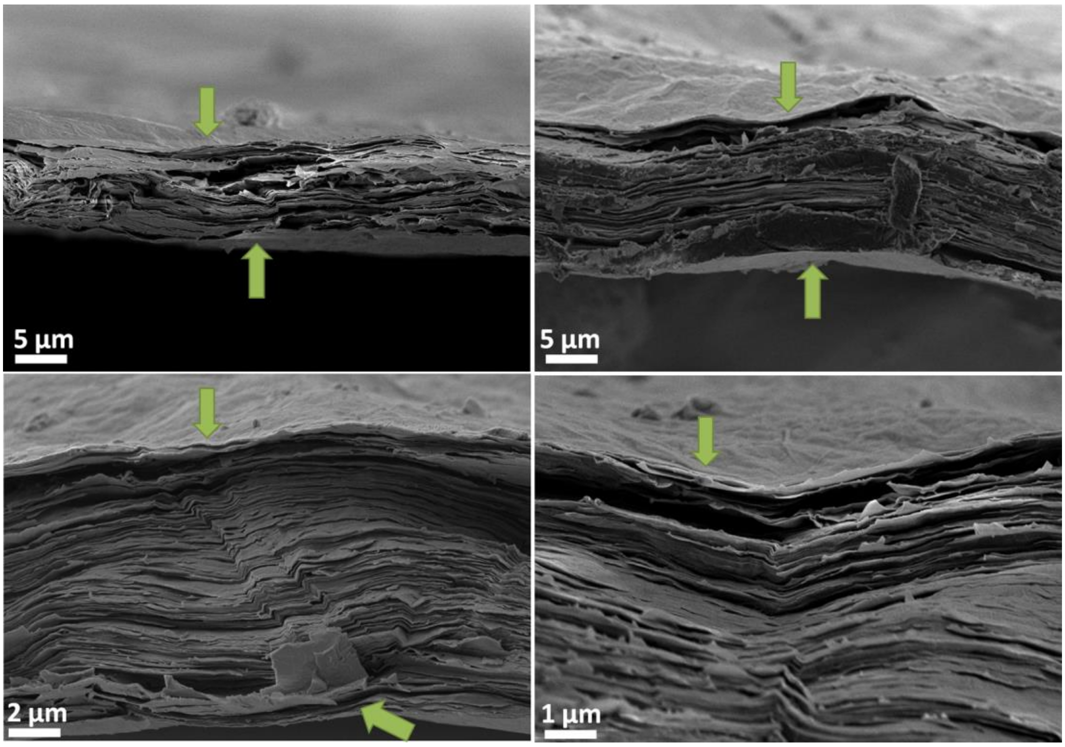

Figure S9 SEM images of the skin-wrinkles-skin structure observed in the C-GOFs.

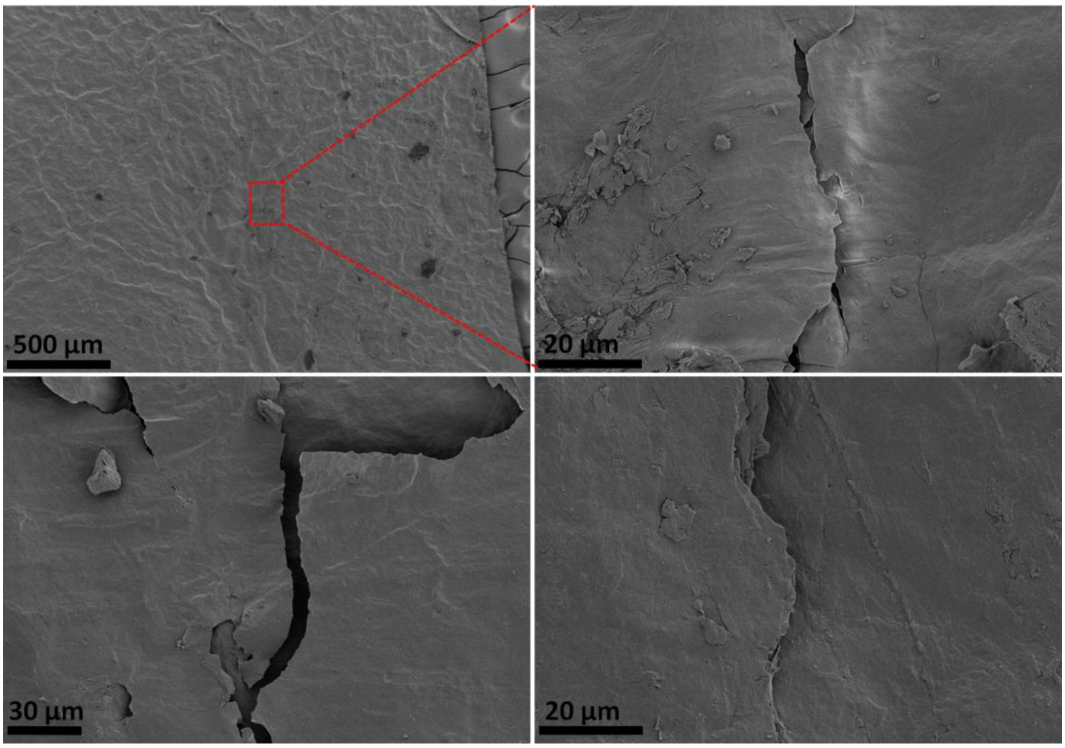

Figure S10 SEM images of the cracks in the skins of the stretched, but not fractured C-GOFs.

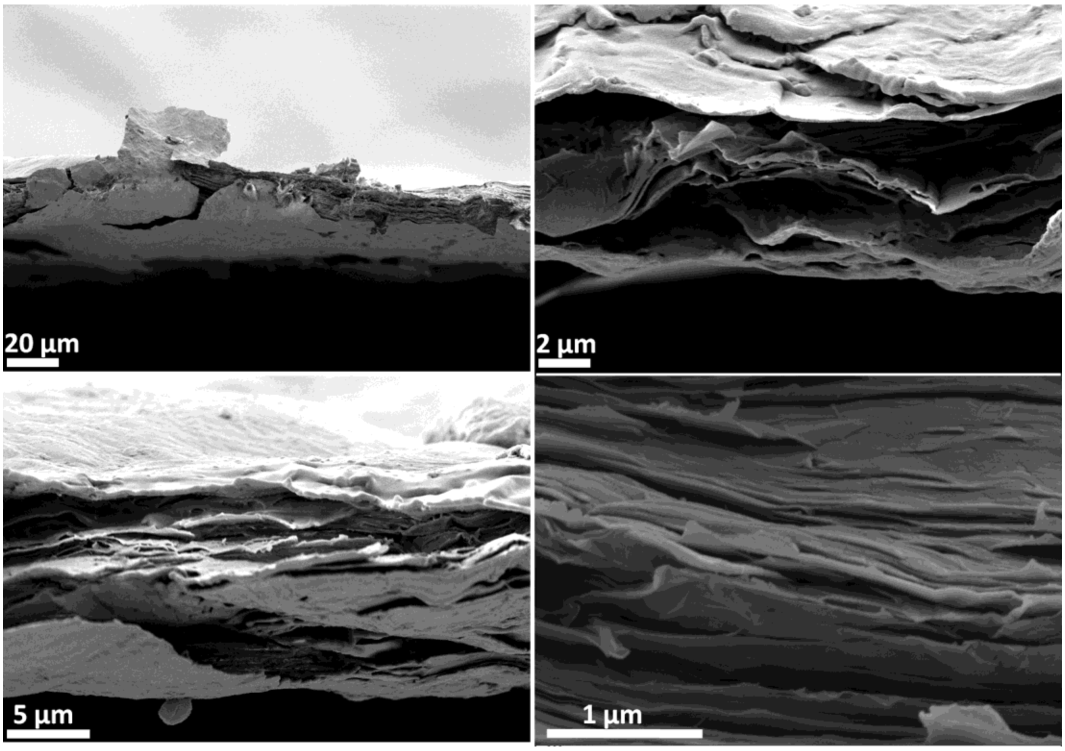

Figure S11 SEM images of the cross-sections of the fractured C-GOFs, which exhibit irregular interfaces.

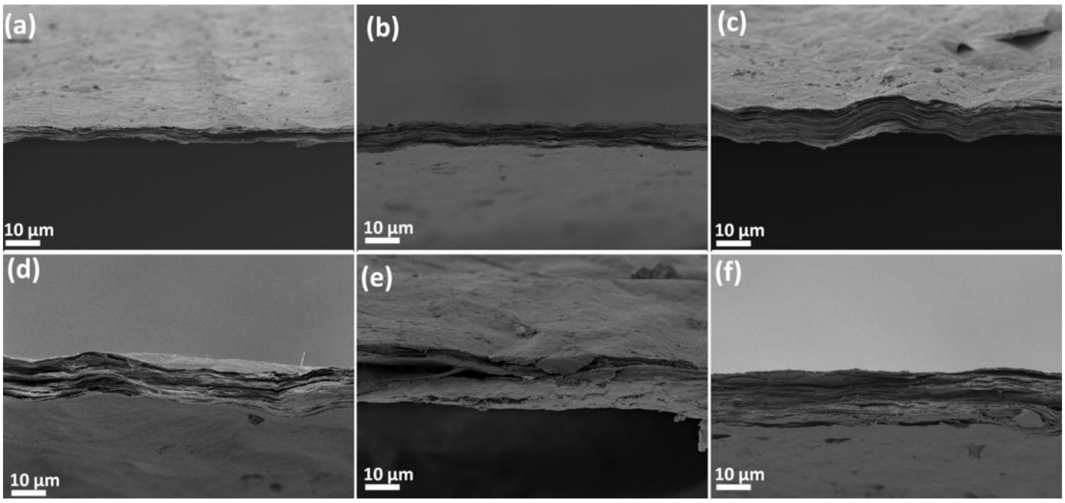

Figure S12 SEM images of the cross-sections of the C-GOFs in different thicknesses.

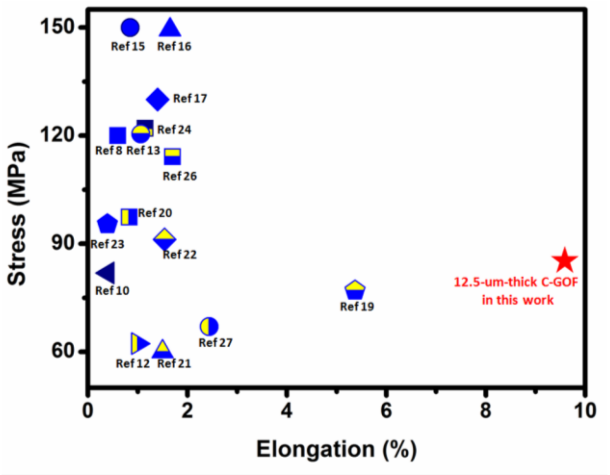

Figure S13 Comparison of tensile strength and elongation of our 12.5-μm-thick C-GOF with those of the reported pure GOFs.

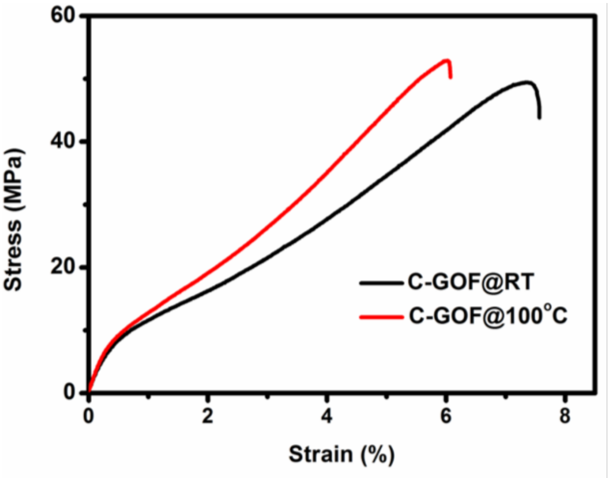

Figure S14 Typical strain-stress curves of the as-prepared C-GOFs, and thermally annealed C-GOFs.

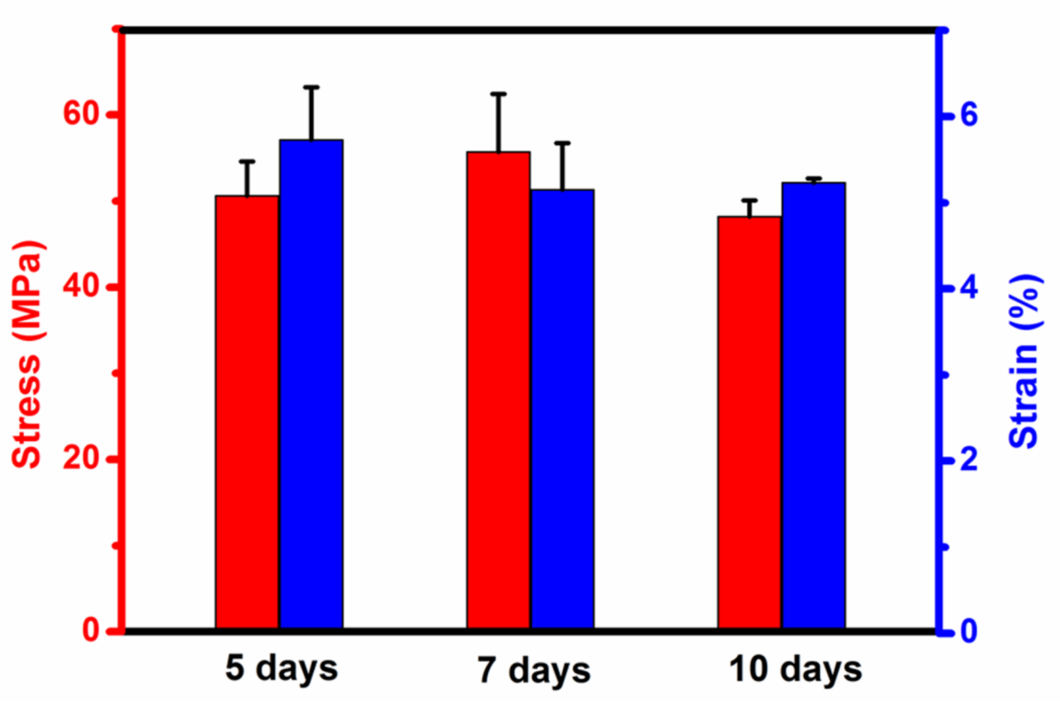

Figure S15 Mechanical properties of the GOFs air-dried for 5, 7, and 10 days. The thickness of these GOFs were around 11 μm.

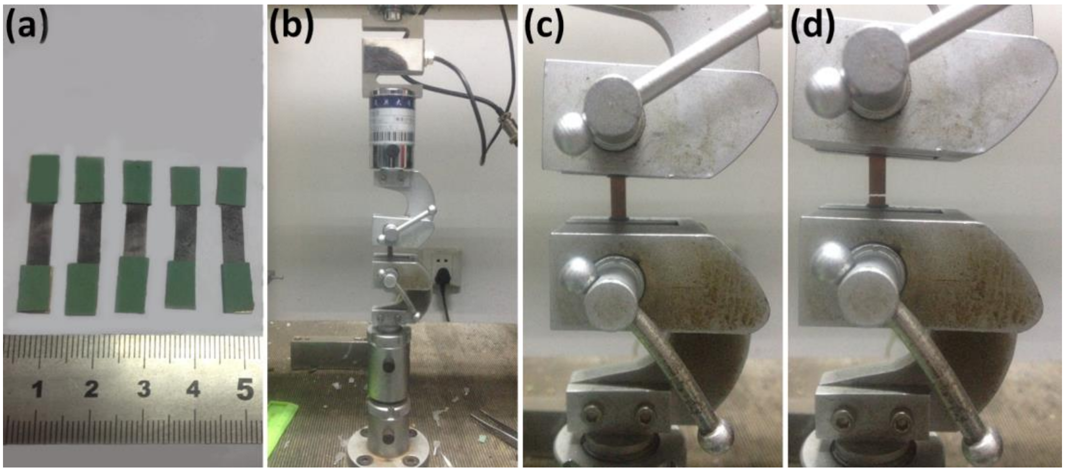

Figure S16. Digital pictures of the GOFs strips (a), set-up (b), and the loading process (c, d).

Table S1 Statistical results of the mechanical properties of the reported pure GOFs.

Assembly method	Tensile strength [MPa]	Elongation at break [%]	Toughness [MJ m^{-3}]	References in paper
Vacuum filtration	120	0.6	~0.35	[8]
Vacuum filtration	150	0.85	~0.64	[15]
Vacuum filtration	81.9	0.4	~0.165	[10, 11]
Vacuum filtration	149.4	1.65	~1.24	[16]
Vacuum filtration	130	1.4	~0.91	[17]
Vacuum filtration	62.3	1	0.5	[12]
Vacuum filtration	97.4	0.83	~0.4	[20]
Vacuum filtration	60	1.5	~0.45	[21]
Vacuum filtration	95.4	0.39	~0.19	[23]
Cast drying	67	2.44	~0.82	[28]
Cast drying	114.1	1.7	~0.97	[27]
Cast drying	91.2	1.54	~0.7	[22]
Cast drying	122	1.15	~0.7	[24]
Cast drying	77	5.37	2.26	[19]
Wet-spinning	120.5	1.06	~0.64	[13]

Table S2 Mechanical properties of F-GOFs and C-GOFs in different thicknesses.

Samples	Average thickness [μm]	Elongation at break [%]	Tensile strength [MPa]	Toughness [MJ m^{-3}]
F-GOF	4.9±0.11	2.46±0.30	56.7±2.3	0.62±0.015
F-GOF	9.4±0.025	3.85±0.40	60.9±2.6	1.42±0.36
C-GOF	4.8±0.2	3.22±0.60	59.6±16	0.78±0.17
C-GOF	7.6±0.2	3.90±0.28	78.5±7.7	1.66±0.19
C-GOF	10.5±0.3	4.15±0.46	62.6±5.3	2.12±0.41
C-GOF	12.5±0.5	9.80±1.90	84.5±9.8	4.37±0.94
C-GOF	13.8±0.5	8.17±0.71	71.2±11	3.46±0.73
C-GOF	15.7±0.4	7.02±0.49	70.8±5.1	3.23±0.53

Table S3 Statistical results of the mechanical properties of the modified GO/rGO-based paper-like materials previously reported.

GO/rGO-based paper-like materials	Tensile strength [MPa]	Elongation at break [%]	Toughness [MJ m^{-3}]	References in paper
MoS_2/rGO	7.8	2.1	~0.084	[37]
Borate/GO	185	0.15	~0.14	[17]
PAM/GO	91.9	0.32	~0.15	[11]
PEI/GO	209.9	0.4	~0.23	[20]
GA/GO	149.4	0.184	~0.3	[12]
Ca^{2+}/GO	125.8	0.5	~0.31	[10]
PVA/GO	118	1.1	~0.71	[28]
rGO	293.3	0.85	~1.25	[15]
PDA/GO	175	1.5	1.5	[22]
Silk/GO	221	1.8	~2	[27]
PMMA/GO	148.3	3.17	~2.35	[16]
Silk/GO	300	1	~2.4	[38]
PCDO/GO	129.6	5	2.52	[23]
Silk/GO	153	2.8	2.6	[21]
Silk/rGO	300	1.8	~2.8	[21]
PAPB/GO	120.5	1.06	3.56	[24]
PCDO/rGO	156.8	8	3.91	[23]
PDA/rGO	204.9	5	4	[22]
PVA/rGO	157	5.6	6.1	[14]
PAPB/rGO	382	4.3	7.5	[24]
